# The evolution of intergroup cooperation

**DOI:** 10.1098/rstb.2022.0074

**Published:** 2023-04-10

**Authors:** António M. M. Rodrigues, Jessica L. Barker, Elva J. H. Robinson

**Affiliations:** ^1^ School of Biology, University of St Andrews, St Andrews, KY16 9TH, UK; ^2^ Schools of Medicine and Engineering, Stanford University, Stanford, CA 94305, USA; ^3^ Department of Ecology and Evolutionary Biology, Yale University, New Haven, CT 06511, USA; ^4^ Surgo Ventures, Washington, DC 20036, USA; ^5^ Interacting Minds Centre, Aarhus University, 8000 Aarhus, Denmark; ^6^ Division of Population Health Sciences, University of Alaska Anchorage, Anchorage, AK 99508, USA; ^7^ Department of Biology, University of York, York YO10 4LZ, UK

**Keywords:** intergroup cooperation, intergroup conflict, coalitions, peace, dispersal, kin selection

## Abstract

Sociality is widespread among animals, and involves complex relationships within and between social groups. While intragroup interactions are often cooperative, intergroup interactions typically involve conflict, or at best tolerance. Active cooperation between members of distinct, separate groups occurs very rarely, predominantly in some primate and ant species. Here, we ask why intergroup cooperation is so rare, and what conditions favour its evolution. We present a model incorporating intra- and intergroup relationships and local and long-distance dispersal. We show that dispersal modes play a pivotal role in the evolution of intergroup interactions. Both long-distance and local dispersal processes drive population social structure, and the costs and benefits of intergroup conflict, tolerance and cooperation. Overall, the evolution of multi-group interaction patterns, including both intergroup aggression and intergroup tolerance, or even altruism, is more likely with mostly localized dispersal. However, the evolution of these intergroup relationships may have significant ecological impacts, and this feedback may alter the ecological conditions that favour its own evolution. These results show that the evolution of intergroup cooperation is favoured by a specific set of conditions, and may not be evolutionarily stable. We discuss how our results relate to empirical evidence of intergroup cooperation in ants and primates.

This article is part of a discussion meeting issue ‘Collective behaviour through time’.

## 1. Introduction

Relationships within social animal groups can be very mixed, with both aggression and cooperation occurring widely. By contrast, relationships between groups of animals are typically concentrated at the conflict end of the conflict–cooperation spectrum [1]. Animal groups compete with, fight, or at best avoid and tolerate other groups. Active transfer of benefits between members of groups that have distinct and separate identities can occur, but this form of intergroup cooperation appears to be restricted to only a few taxa, primarily humans, bonobos and some ants [2,3]. Here, we ask why intergroup cooperation is so rare, and under what conditions it can evolve.

Here, we define cooperation as ‘the transfer of benefits from one party to another, ultimately resulting in direct or indirect fitness benefits to both parties' [2]. We contrast this with tolerance, in which one party may either benefit or at least incur no costs and the other incurs neither costs nor benefits, and also with conflict, in which one or both parties incur costs [2]. We can apply these definitions of positive, neutral and negative relationships to within-species intergroup interactions, defined as the reciprocal action or influence of multiple groups on each other [2], where a group is broadly defined as a spatial association of interacting conspecific individuals that is stable over the timescale of the interactions of interest.

Two core potential drivers of intergroup cooperation have been proposed: overarching threats from predators, competitors or adverse conditions, and group-level asymmetries in either type or quantity of resources [2]. These challenges provide potential adaptive benefits to intergroup cooperation: defence against threat, or resource transfer. While adaptive benefits such as these are *necessary* for intergroup cooperation to evolve, they are not *sufficient* to explain why intergroup cooperation evolves only in certain, rare, circumstances. Neither is invoking kinship *sufficient* to explain the patterns of intergroup relationships. In the case of individuals, cooperating with kin reaps inclusive fitness benefits, but these benefits can be cancelled out by increases in local kin competition [4]. Similarly, if groups are interrelated, because of local dispersal or formation through fission, then competition between groups can cancel out the kin-selected benefits of cooperation with neighbouring groups [5].

Under what conditions, then, might we expect intergroup cooperation to arise? The most intuitive reason for intergroup cooperation to occur is in order to accrue the benefits of enlarged group size. Increased group size can provide protection against predators, competitors and environmental conditions [6,7], and temporary fusion of multiple groups is a common response to such challenges [8]. In these cases, there may be a hierarchical group structure, with tighter cooperation within subgroups, usually family units, and a looser tolerance between the groups that have temporarily fused. This reduction in aggression within the expanded group may reduce dispersal by lowering the costs of staying in or near the natal group but, in the longer term, local competition would be expected to reduce the benefits of this tolerance [5]. It is unclear when defence against external threats should provide selective pressure that would lead to stable intergroup cooperation, in the form of the exchange of benefits across group boundaries, as opposed to generating a public good in the form of a large group size. The model we present here explores circumstances under which this form of intergroup cooperation can evolve.

The second core potential driver of intergroup cooperation is the resource environment, but its impact on cooperation is somewhat more complex. Scarcity of resources typically promotes within-group competition, but is likely to lead to conflict with outgroups [1,9,10]. Cooperation between groups is thus most likely when either (i) resources are accessible only through collaboration between multiple groups or (ii) resources are sufficiently plentiful that the benefits of competing for additional resources are outweighed by the costs of conflict. An environment where resources are stable and relatively abundant is likely to promote tolerance between groups but would not itself promote a transition to active cooperation between those groups. We suggest that this transition can come about when there is spatial or temporal heterogeneity within an environment of relative abundance. Temporary resource asymmetries provide scope for intergroup cooperation, as they create a supply–demand imbalance that can be corrected by relatively low-cost resource transfer. In some highly related societies, such as ‘polydomous’ ant colonies (colonies spread between many spatially separate but socially connected nests [11]), resource redistribution from oversupplied to undersupplied groups can indeed occur in response to such local resource asymmetries [12,13]. In human societies, however, inequality in resource availability does not necessarily promote cooperation [14,15]. Indeed, in most animals, such intergroup resource transfer would be unexpected, and yet it has been observed in bonobos, where high-value food, meat, can be shared with individuals from neighbouring groups [3]. What evolutionary pre-conditions could lead to the scenario where resource transfer between groups can evolve?

We outline a hypothesized set of evolutionary stages in table 1, through which intergroup cooperation could develop, illustrated with examples of primates and social insects. (1) A group exploits large (and predictably large) resources, such that competition for food among individuals within the group is reduced. Group size is likely to increase. (2) Groups can dominate resources by employing behavioural mechanisms to reduce intragroup competition for food. (3) The costs of competing with kin are potentially offset by the benefits of excluding other less related groups, allowing reduced dispersal, and the emergence of kin-structured populations. (4) The reduction in intragroup aggression could then extend to between neighbouring (related) groups. Many fission–fusion systems could be seen to operate in this way, or groups could switch between this state when resources are abundant and a more fragmentary state when they are rare. (5) This intergroup tolerance provides an environment in which more advanced cooperation, e.g. resource or information transfer between groups, could evolve, given the right parameters. Steps 1–4 are contingent on a relatively stable large key resource; the transition to step 5 is likely to emerge only if there are secondary spatially and or temporally heterogeneous resources.

**Table 1.** Hypothesized stages of development of intergroup cooperation over evolutionary time.

	polydomous ants	bonobos
1. Large stable resources; increased group size	aphid honeydew (from trees or generalist across many plants) for large, stable and predictable carbohydrate source [16]	terrestrial herbaceous vegetation: dense, stable and predictable food source [17]
2. Reduced intragroup aggression; exclusion of competitors	nest-mate recognition, low within-group aggression [18,19]	socio-sexual behaviour, low within-group aggression [17,20]
3. Reduced dispersal	secondary polygyny and dispersal by budding rather than flight [19]	evidence of relatedness between neighbouring groups suggests reduced dispersal [21,22]
4. Reduced intergroup aggression	high genetic and chemical similarities between neighbouring groups result in lowered aggression [19]	intergroup aggressive displays may occur, but rarely result in actual injury [23]
5. Intergroup resource sharing (food, information)	resource transfer from successful nests to temporarily less successful nests, evening out spatial heterogeneity [12,13]	meat-sharing at borders between groups [3], more efficient foraging in unfamiliar areas associated with intergroup tolerance [24]

Here, we use a mathematical model to test key steps in the evolution of intergroup cooperation. We test whether the threat of intraspecific competition can provide a credible driver for intergroup cooperation, and under what conditions we might expect this to arise. Our model allows inter- and intragroup conflict and cooperation and integrates ecological and genetic drivers of social interactions. Our model formalizes the dispersal and movement mechanisms that underlie group formation, as well as the multi-level social structure of the population, and how the different social units interact with each other.

## 2. Methods

### (a) Modelled life cycle

**Figure 1.**
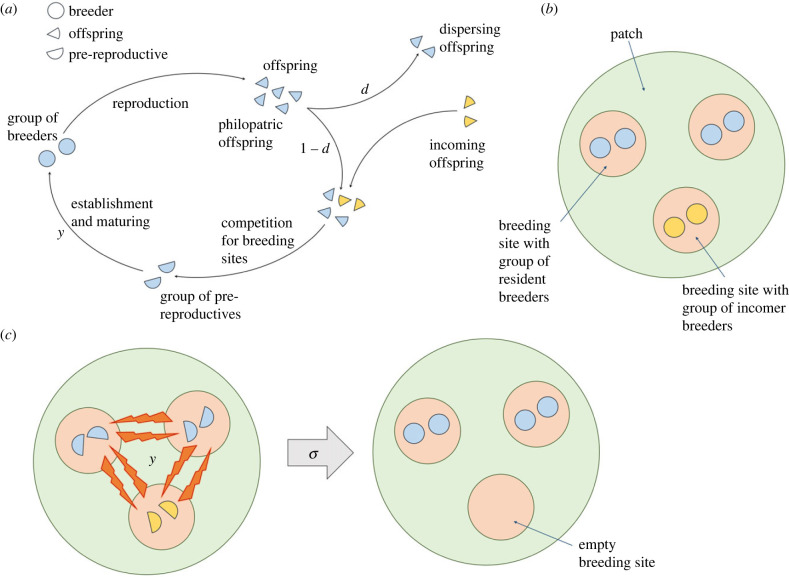
Conceptual overview of model. (*a*) Life cycle of modelled organism; (*b*) example of a fully occupied patch. Here groups are shown with size 2, but the model allows for any size group. (*c*) During the establishment and maturation phase, groups of pre-reproductives interact with probability *y*, and if they interact, one group is eliminated with probability *σ*.

We assume an infinite population with multiple levels of social organization, in which the population is subdivided into patches, and individuals within each patch are subdivided into groups [5]. Groups are composed of resident individuals, if they were born in the local patch, or incoming individuals, if they were born elsewhere (figure 1*a*,*b*). We assume that all patches initially contain three groups, and each group, either resident or incomer, contains *n* pre-reproductive individuals. Interactions between the three groups can occur, and can result in the disruption of one group of pre-reproductives (figure 1*c*). In particular, we assume that, with probability *y*, intergroup interactions are initiated (an ‘attack’ by two dominant-acting groups against a subordinate-acting group). With probability *σ*, these interactions result in the successful disruption of one group and the survival of the other two groups. Otherwise, with probability 1 − *σ*, the attack is unsuccessful in that the dominant groups may pay a cost. Below, we provide a detailed description of the social interactions among groups and the costs of unsuccessful attacks. After intergroup interactions, each surviving group establishes itself as a group of breeders, and each group member produces a very large number *f*_i_ of offspring. If some groups are eliminated during intergroup interactions, the additional resources may benefit the fertility of the surviving individuals, which are then able to produce *f*_i_ (> *f*_0_) offspring, where ‘*i*’ is the number of groups that were eliminated. A fraction 1 − *d* of the offspring remains in the native patch, while a fraction *d* disperse to a random distant patch. In addition to long-distance dispersal between patches, we also allow the movement of offspring between groups [5]. In particular, we assume that a fraction 1 − *m* of those offspring that remain in the native patch also remain in the native group, while a fraction *m* move at random to one of the two other groups in the focal patch. We assume that long-distance dispersal carries a cost *c*, such that only a fraction 1 − *c* of the dispersed offspring survive and arrive at a foreign patch, while—for simplicity of analysis—local movement between breeding areas is costless. The dispersed offspring, after arriving at a patch, attempt to obtain resources in one of the three breeding areas. Offspring within each breeding area form groups of *n* individuals. We assume that patch-philopatric offspring always group together with other patch-philopatric offspring, while incoming offspring always group together with other incoming offspring, such that groups are exclusively formed by either patch-philopatric offspring or by incoming offspring. This stage brings the life cycle to its starting state, and the life cycle resumes (figure 1*a*). See electronic supplementary material for more details.

### (b) Intergroup interactions

We consider a scenario in which two groups may disrupt a third group. Underlying our scheme of social interactions is the idea that groups of philopatric individuals have a home-ground advantage. When incoming groups are present, the group that faces the threat of being disrupted and eliminated (subordinate group) is always an incoming group. If all groups in a patch are resident, and therefore incoming groups are absent, then all groups have the same probability of being the subordinate group. When two or more incoming groups are present, each group is equally likely to be the subordinate group. Thus, when a patch contains three of the same kind of group (all philopatric or all incomer), two of the groups attempt to disrupt the third group, and all groups are equally likely to be in one of the two situations.

For all cases, the two dominant-acting groups attempt to disrupt the third group with probability *y*. The attempt is successful with probability *σ*, in which case the two dominant groups establish as reproductively active, i.e. as groups of breeders (figure 1). With probability 1 − *σ*, the attempt is not fully successful, and the dominant groups may pay a cost. The cost of intergroup disruption depends on the behaviour, which may take two forms. First, we consider a cooperative form of behaviour in which the two dominant groups share the same fate: either their attempt to disrupt the third group is successful, and they both survive, or the attempt is unsuccessful, and they both die (electronic supplementary material, SC). Second, we consider an altruistic form of behaviour in which the two dominant groups may have distinct fates: either their attempt to disrupt the third group is fully successful, and they both survive, or the attempt is only partially successful, and one disrupting group dies, while the other accrues the benefits (electronic supplementary material, SD). In both cases, the phenotype *y* is conditionally expressed on the condition of the dominant groups, either resident or dispersed, and on the number of resident and incoming groups in the focal patch. We write *y_η_*_,*α*_ to denote the phenotype of a condition-*η* individual, either resident or incoming, in a patch with *α* resident groups (see electronic supplementary material, SB–SD for details). While our model assumes that tolerance preferentially occurs among resident groups, and these are likely to be related, we do not assume an explicit mechanism of kin discrimination. Rather, other cues or asymmetries between resident and incoming groups underlie the mechanisms that lead to tolerance between groups.

### (c) Analysis

To analyse the model, we take the neighbour-modulated approach to kin selection [25,26], and we employ the concept of class-structure to define the social structure of the population [5], as well as the condition of individuals, which includes the resident–incomer dichotomy, and the number of groups in a patch [27]. We start by defining the neighbour-modulated fitness for each class of individuals, and we then derive the selection gradient for the trait of interest *y*. We present the selection gradient in terms of the inclusive fitness effect, which partitions selection into a direct component—the effect of an individual's behaviour on its own fitness—and an indirect component—the effect of an individual's behaviour on the fitness of social partners. In the electronic supplementary material, we provide a detailed description of the methodology.

## 3. Results

### (a) Inclusive fitness effect

We start by outlining the inclusive fitness effect of the shared costs form of behaviour, in which the two groups that stand to gain from the intergroup interactions, the dominant-acting groups, share equally both costs and benefits of engaging in intergroup interactions. Employing the methodology described above (see electronic supplementary material for details), we find that the inclusive fitness effect is given by

3.1 $$\Delta w_{\mathrm{IF}}=[1+(n\text{–}1)r_{\alpha }^{w}](\sigma v_{\beta }\text{–}v_{\alpha })+nr_{\alpha }^{b}(\sigma v_{\beta }\text{–}v_{\alpha })\text{–}nr_{\alpha }^{t}v_{\alpha },$$

where: $r_{\alpha }^{w}$ is the relatedness between the focal actor and group mates; *v*_α_ is the reproductive value of a focal individual in the absence of social interactions; *v_β_* is the reproductive value of a focal surviving individual if the attack is successful, and $r_{\alpha }^{b}$ is the relatedness between the focal actor and an individual in the other aggressor group. The right-hand side of equation (3.1) describes the inclusive fitness effect of the behaviour. First, by engaging in conflict, the attacking groups forego the reproductive value *v_α_* associated with the current social state of the patch (the composition of the patch in terms of resident versus incoming groups), which we denote by *α*. With probability *σ*, the attack is successful, in which case the attacking groups eliminate the third group and generate a reproductive value *v_β_*, where *β* denotes the state of the patch after the successful attack. These fitness effects impact all individuals in the patch. They affect the focal individual, which is related to herself by 1, but also the *n* − 1 group partners of the focal individual, which are related to the focal individual by $r_{\alpha }^{w}$. Second, the behaviour also affects the *n* individuals in the other attacking group, who are related to the focal individual by $r_{\alpha }^{b}$. Finally, the attack causes the loss *v_α_* in the reproductive value of the *n* individuals of the third group, whose group members are related to the focal individual by $r_{\alpha }^{t}$.

Let us turn our attention to the inclusive fitness effect of the altruistic form of behaviour, in which one of the aggressor groups dies, allowing the survival of the other dominant group and elimination of the third group. In such case, we find that the inclusive fitness effect is given by

3.2 $$\begin{array}{rl} \Delta w_{\mathrm{IF}} & =(1+(n\text{–}1)r_{\alpha }^{w})(\sigma v_{\beta }\text{–}v_{\alpha })+nr_{\alpha }^{b}(\sigma v_{\beta }+(1\text{–}\sigma )v_{\delta }\text{–}v_{\alpha })\\ & \;\text{–}nr_{\alpha }^{t}v_{\alpha }, \end{array}$$

where *v_δ_* is the reproductive value of surviving individuals in the second dominant-acting group when both the focal altruistic group and the third group die, and where *δ* represents such a patch state. If we compare the selection gradient in equation (3.1) with the selection gradient in equation (3.2), we find that they are nearly identical, except for the term (1 − *σ*)*v_δ_*. This term affects the *n* individuals in the surviving group only, because it emerges from the self-sacrificing behaviour of the first dominant group. That is, with probability 1 − *σ*, both the first dominant group and the third group die, in which case the individuals in the second dominant group generate a reproductive value *v_δ_*.

### (b) The evolutionary ecology of intergroup interactions

#### (i) Dispersal and social context

**Figure 2.**
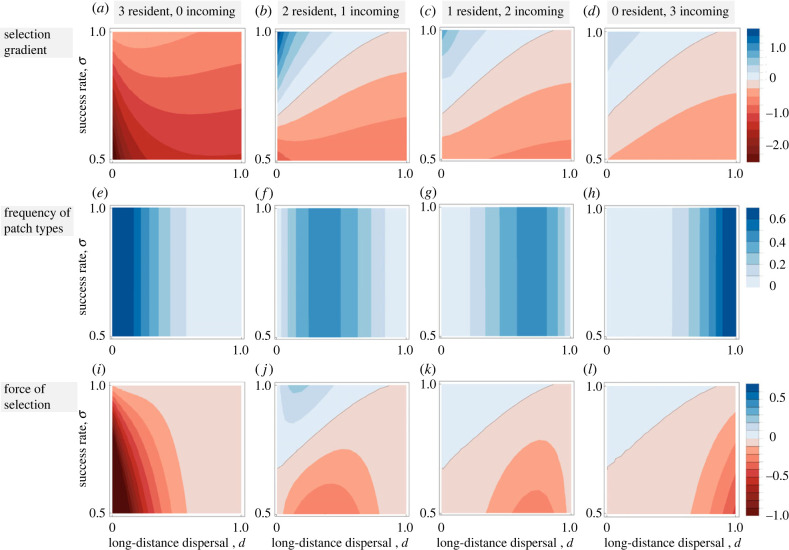
Selection gradient, frequency of patch types and force of selection as a function of long-distance dispersal, *d*, and the success rate of disruption, *σ*, for different types of patches. In patches with three resident groups, disruption is disfavoured because all groups are related to each other. In all other patch types, disruption may be favoured when dispersal is low and success rate is high. Disruption is most favoured in patches with two resident groups and one incoming group, because the two resident groups are related to each other but unrelated to the incoming group, and because the frequency of these patches is high for the range of parameter values where disruption is favoured. Parameter values: *m* = 0.6, *c* = 0.1, *n* = 2.

When disruption of a third group frees up a breeding area for offspring of the other two dominant-acting groups, then disruption is more likely to evolve when these two dominant groups are both resident groups and their target is an incoming group (figure 2*a–d*). There is selection for a pattern of intergroup interactions where two groups that tolerate each other attack a third group both in patches with two resident groups and in patches with one or no resident group. In all three cases, intergroup aggression is more likely when the success rate of attacks is high (figure 2*b*–*d*). In addition, intergroup aggression is more strongly selected when long-distance dispersal is relatively lower. However, when dispersal is lower, the frequency of patches with one or no resident group(s) is significantly lower than the frequency of patches with two resident groups (figure 2*e*–*h*), and therefore overall force (or intensity) of selection (cf. [27]) for intergroup aggression is stronger in patches with two resident groups (figure 2*i*–*l*). In addition, relatedness also favours the evolution of intergroup aggression in patches with two resident groups, as intergroup aggression can generate indirect fitness benefits both through effects on group mates and through effects on individuals of other groups. When at least one of the attacking groups is an incoming group, both relatedness within incoming groups and relatedness between groups is zero, in which case indirect fitness benefits are null, which, in turn, weakens selection for disruption.

#### (ii) Fecundity versus survival benefits

**Figure 3.**
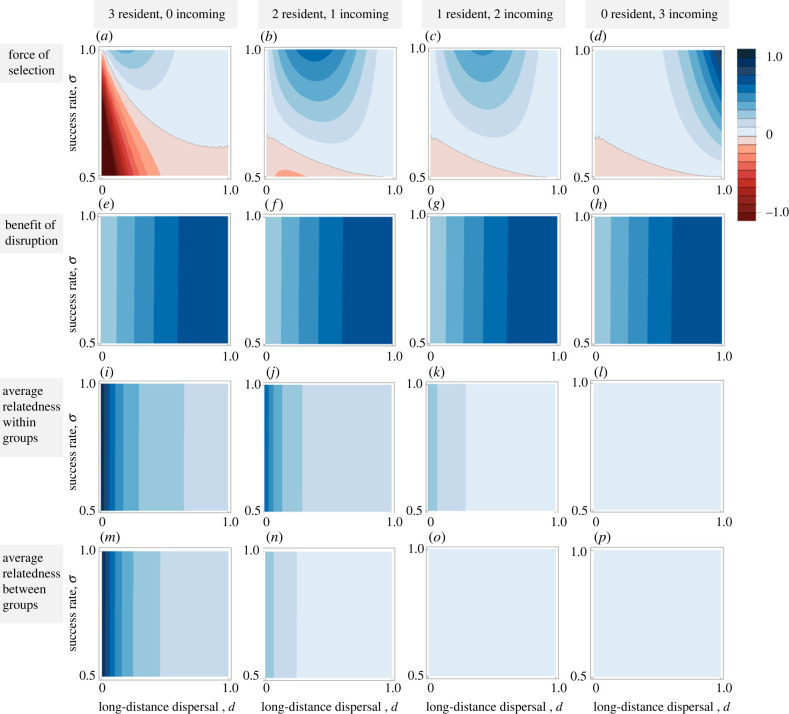
Force of selection, fitness benefit of disruption, average relatedness within groups and average relatedness between groups as a function of long-distance dispersal, *d*, and the rate of disruption, *σ*, for different patch types, assuming fertility benefits. When disruption of a neighbouring group generates fertility benefits, the fitness benefits of disruption increase with dispersal. In such cases, direct fitness benefits outweigh indirect fitness benefits, and disruption is most favoured in patches with three (unrelated) groups when dispersal is high. High dispersal allows mothers to export the additional fertility benefit, and therefore avoid competition among kin offspring. Parameter values: *c* = 0.1, *f*_0_ = 1, *f*_1_ = 2, *n* = 2.

Above, we have considered a scenario in which social interactions can free up breeding sites, which improves offspring survival, but does not affect the fecundity of adult breeders. Here, we consider the scenario in which social interactions that disrupt a third group can benefit the fecundity of surviving groups. We find that in such cases, a pattern of intergroup interactions where two groups tolerate each other while attacking a third group can evolve even when a patch contains three resident groups (figure 3*a*). By contrast with the survival-benefits-only scenario (figure 2*a*–*d*), selection for intergroup aggression is now stronger under higher levels of long-distance dispersal (figure 3*a*–*d*). Fecundity benefits mean that surviving individuals produce more offspring. If long-distance dispersal is lower, the additional offspring also impose extra competition on their kin, which tends to offset the fecundity benefits of intergroup aggression (figure 3*e–h*). By contrast, if long-distance dispersal is higher, most of the additional offspring disperse, and therefore avoid the costs of kin competition (figure 3*i–p*). However, when dispersal is high, the frequency of patches with two or more resident groups decreases rapidly, which weakens the force of selection operating in these patch types (figure 3*a*–*d*).

#### (iii) Form of behaviours and movement between groups

**Figure 4.**
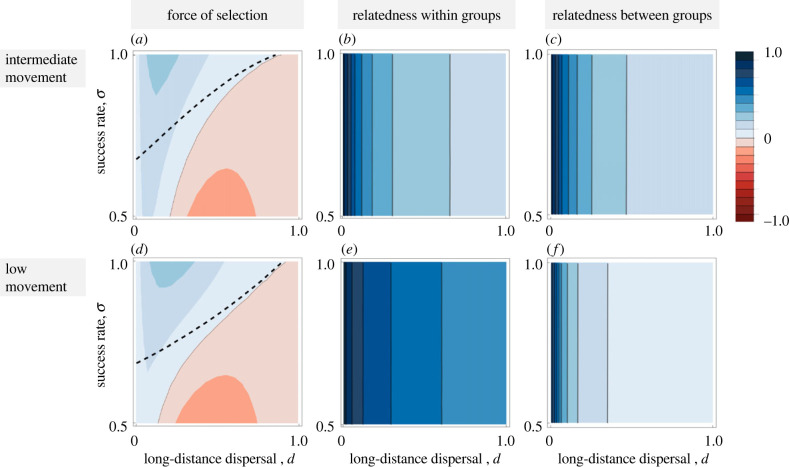
Force of selection, relatedness within groups, and relatedness between groups as a function of long-distance dispersal, *d*, and the success rate of disruption, *σ*, for intermediate movement between local groups (*m* = 0.6) and for low movement between local groups (*m* = 0.1) in patches with two resident groups and one incoming group. Within the region above the dashed line in (*a*) the behaviour is cooperative, while below the dashed line the behaviour is altruistic. Little movement between local groups increases relatedness within groups but reduces relatedness between groups. By contrast, intermediate movement between groups equalizes relatedness within and between groups, which tends to favour altruism between groups, especially when rates of disruption are relatively low. Parameter values: *c* = 0.1, *n* = 2. (Online version in colour.)

We have considered behaviours in which the costs and benefits are shared among the two dominant-acting groups. Here, we contrast these shared-cost behaviours with an altruistic form of behaviour in which one of the dominant groups effectively engages in altruistic self-sacrificing behaviour allowing the second dominant group to survive. We find that selection acting on these forms of behaviour is sensitive to the degree of movement between groups (figure 4*a*–*d*). In particular, selection acts less strongly on altruistic behaviours when movement between groups is limited. Altruistic behaviours require high relatedness between groups. When movement between groups is limited, relatedness between groups is lower, and individuals generate little indirect fitness benefit (figure 4*d*–*f*). By contrast, relatedness between groups is highest when movement between groups is intermediate. In such cases, if a group dies, their members can still generate fitness through the indirect component, as long as the second non-sacrificing group survives (figure 4*a*–*c*). Note that because altruistic behaviours require relatedness between groups, these forms of behaviour can only evolve in patches that contain at least two resident groups.

## 4. Discussion

Our model demonstrates that multi-group interaction patterns, in the form of aggression towards one group combined with tolerance of another, can be favoured by evolution under certain circumstances and can result in cooperative participation in intergroup conflict. In a viscous population, where long-distance dispersal is low, we find that these results can extend to altruistic participation in intergroup conflict owing to the build-up of relatedness between groups. In general, the evolution of these intergroup interactions is influenced strongly by the population viscosity, and by the nature of the costs and benefits of the intergroup interactions. Our evolutionary model thus provides a possible route by which intergroup cooperation could arise.

When the benefits of group elimination are accrued by the next generation in terms of increased offspring success in the scramble competition for new breeding sites, then intergroup interactions (disruption of one group by the other two) are favoured by lower levels of dispersal. These behaviours are, however, most beneficial in mixed patch types, where it is the incomer groups that are most likely to be eliminated. At very low levels of dispersal, patches become occupied only by residents, and both mixed patches and the benefits of these intergroup behaviours disappear.

Our model assumes that in mixed patches, incomer groups are more likely than resident groups to succumb to intergroup disruption, but this does not require active targeting of incomer groups. Ecologically, we expect resident groups to have an advantage over incomer groups because residents save time and energy by not dispersing, which they can invest in growth and resource acquisition. We also do not assume the ability to recognize neighbouring groups as kin: in its simplest case, the preferential elimination of incomer groups in mixed patches could be an emergent outcome of all groups attempting to disrupt all others, but incomers being less well-equipped to defend themselves. Indiscriminate conflict is costly, however, and evolution favours strategies for avoiding costly conflicts [5,28,29], so we might expect targeting mechanisms to evolve, for example, preferential attacks on smaller groups [30,31]. This could still result in effective preferential elimination of incomer groups, if resident groups can grow more quickly, because members did not first disperse. A more direct targeting of disruption could be some assessment of familiarity or self-similarity: such mechanisms are commonly used for between-group discrimination [18,32,33], and can also be used to distinguish between members of a meta-group, or supercolony (other resident groups, in our model), and incomers from a different wider group, as seen in some ants [34]. Finally, groups might actually recognize specific individuals and their group memberships, and form active coalitions with individuals from known groups to attack or exclude other individuals [8,35].

Our model predicts that these intergroup behaviours are not favoured in low-dispersal resident-only populations. Certain polydomous ant species have lost long-distance dispersal ability entirely, and form new nests only by local budding. In these species, remarkable tolerance of neighbouring groups can be observed [2,13,36]. Our model predicts that such tolerance should be maintained only if there is a possibility that incomer groups might arrive; in that event, the mutually tolerant groups can cooperatively eliminate potential competitors [37].

The nature of the benefits arising from intergroup interactions strongly influences the evolution of these behaviours. When the benefit is increased offspring survival in the next generation, as discussed above, these intergroup interactions are favoured by lower dispersal. By contrast, this pattern is reversed when the resources freed up by elimination of a group can be acquired by the other groups present in a patch and used to increase fecundity (compare figures 2 and 3). This is because the benefit of increasing the number of offspring produced will be offset by the costs of kin competition among those offspring, unless those offspring disperse [5,38]. The nature of the costs of intergroup interactions will also influence the evolution of these behaviours. We assume that if interactions are unsuccessful, the cost of the conflict means that groups are unable to establish breeding groups and produce offspring. In many ecological scenarios, however, these costs will be less steep, in which case we expect natural selection to favour intergroup cooperation in aggression across a wider range of parameter values; this scenario does not change our observed relationship between dispersal and intergroup cooperation (electronic supplementary material, SH1).

Whether fecundity or offspring survival, or both types of benefit, are affected by intergroup interactions will depend on the nature of the resource environment and species ecology. At a coarse level, we would expect intergroup cooperation of the sort seen here (where two resident groups effectively abstain from attacking each other, while attempting to eliminate a third) only when resources are sufficiently abundant to support at least two groups. Otherwise, the kin-selected benefits of preferentially helping one's own group within a patch would dominate, especially if within-patch movement is low, so many offspring stay at the breeding site where they were born. Adequate stable resources (table 1) would seem a prerequisite for this relaxation of intergroup competition; indeed, a resource-focused spatial model of intergroup interactions in early humans finds that sharing food between groups is beneficial to survival when resources are moderately plentiful [39]—when resources are very plentiful, there is little benefit to sharing, and when they are scarce, the costs of sharing are too high. Depletion of resources can lead previously stable cooperation to rapidly break down [40,41]. At a finer scale, groups many require many different resources which may be heterogeneously distributed in space, and once intergroup tolerance has been established, there is the opportunity for groups to develop more active cooperation in the form of the exchange of spatio-temporally patchy resources, as seen in some ants [12] and bonobos [3], and as trade in humans [42].

The costs of engaging in intergroup conflict may not be borne equally, even among those that survive. In our shared-costs model, we assume that the costs are equally shared among those that stand to gain from intergroup disruption. In our asymmetric costs model, we assume that only one of these disrupting groups bears the cost of failure. In mixed patches of two resident groups and one incomer, success would then involve the elimination of only the incomer group, whereas ‘failure’ results in the death of both the incomer and one resident group. This means that the members of one of the resident groups (Group A) engaged in an intergroup conflict from which only the offspring of the remaining resident group (Group B) benefit from two vacant breeding sites in the next generation. This is effectively an altruistic act, in that the members of resident Group A pay a cost and the members of Group B benefit, but this does not require any deliberate engagement in altruism from Group A. In a more complex, natural scenario, the likelihood of such behaviour evolving would depend on the costs of declining to participate, and on the relatedness between the groups, which is strongly influenced in our model by the amount of within-patch movement (figure 4). The relatedness between groups would also be expected to influence the selection pressure for surviving groups to attempt to dominate the resources provided by the elimination of a competitor group. We assume that benefits are shared equally between surviving groups, and as long as intermediate levels of within-patch movement take place, this is likely a stable outcome (electronic supplementary material, SH2). However, under low within-patch movement, resource domination by a single group, if possible, would be favoured.

While we have mostly focused on non-human animals in the set-up and interpretation of our model, there are ways in which our model is applicable to the evolution of intergroup cooperation in humans, particularly for exploiting non-local resources and for buffering resource shortfalls [43]. First, early humans were dependent on resources outside their local region, for example, for making stone tools: although Neanderthals seemed not to travel [44], there is evidence that they used stone brought in from further sites [45]. Second, models based on ethnographic data from hunter–gatherer societies show that collaborations can emerge to exploit specific resources, such as hunting large marine mammals [46,47]. More generally, cooperation in the form of food-sharing is thought to be a key milestone in the transition from primate to human societies [3].

However, there are also notable differences between intergroup cooperation in humans versus non-human animals. In humans, intergroup collaboration seems to have emerged when resources became highly variable and unpredictable [48]. There is also evidence from current small-scale societies that risk-buffering and reciprocal exchange facilitate larger-scale cooperation [49]. In addition, the step of reduced dispersal and kin-based groups applies less to humans than to other animals: early human groups showed dispersal over long ranges and were approximately one-third non-kin [50]. Finally, there are implications of culture in human groups. From around 2 Ma, early humans' ability to use culture to construct niches [51] has complicated how the evolution of intergroup cooperation depends on ecological context. Recent work suggests that the establishment of peaceful intergroup interactions requires cultural institutions such as social norms that dictate non-aggression [52].

The spatial arrangement of groups affects their likelihood of interacting and of experiencing correlated environmental conditions. Our model uses a simplified spatial context, with patches in which groups can interact locally, and between which long-distance dispersal is possible. In reality, both local and longer-range spatial networks are more complex, and groups’ proximity and relative positions within a wider network can have substantial influence on the likelihood of intergroup cooperation in both humans [39,53–55] and ants [56,57]. Our model provides a starting point for more complex spatial structures linking to specific ecological or geographical contexts.

## 5. Conclusion

The evolution of intergroup interaction patterns, including both intergroup aggression and intergroup tolerance or even altruism, is driven by population social structure, and the costs and benefits of intergroup conflict. Local dispersal plays a key role in favouring intergroup cooperation against an incoming enemy group, but as population viscosity increases, encounters with incomers wane, and the benefits of intergroup cooperation likewise decrease. This feedback process means that intergroup cooperation of this kind may not be evolutionarily stable in itself, but under the right ecological conditions, it could provide a baseline of positive intergroup interactions from which other forms of intergroup cooperation could emerge.

## Data Availability

The equations necessary to reproduce the model are provided in the electronic supplementary material [58].
